# Balancing strength and translucency: The role of microstructure in additive and subtractive dental zirconia

**DOI:** 10.1016/j.dental.2025.03.310

**Published:** 2025-04-11

**Authors:** Mirelle M. Ruggiero, Chek Hai Lim, Thomas S. Giugliano, Mijin Choi, Altair A. Del Bel Cury, Yu Zhang

**Affiliations:** a School of Dental Medicine, University of Pennsylvania, 240 S. 40th St., Philadelphia, PA 19104, USA; b University of Campinas, Piracicaba Dental School, Av. Limeira, 901, Piracicaba, SP 13414-903, Brazil; c New York University College of Dentistry, 345 E 24th St., New York, NY 10010, USA

**Keywords:** 3Y-TZP, Milled zirconia, 3D-printed zirconia, Build orientation, Microstructure, Translucency, Strength

## Abstract

**Objectives:**

To elucidate the effect of microstructure on the strength and translucency of dental zirconia fabricated using additive (AM) and subtractive manufacturing (SM) technologies.

**Methods:**

Twelve 3Y-TZP discs were fabricated using AM with two print orientations (0°: group AM0; 90°: group AM90; *n* = 6), and six via CAD/CAM machining (group SM). Density, composition, roughness, translucency parameter (TP), and biaxial flexural strength (σ) were evaluated. Fractographic analysis was conducted and defect size estimated. Based on the preliminary *σ* results (*n* = 6), the optimal print orientation was identified. Nine additional specimens were prepared for each of the AM90 and SM groups for Weibull σ analysis (*n* = 15) Differences in Weibull modulus were assessed via non-overlapping 95 % confidence intervals. An one-way ANOVA followed by Tukey’s post-hoc test and an independent samples t-test were used (α = 0.05).

**Results:**

The relative density was consistent across all groups (>99 %). The tetragonal and cubic phases were comparable among groups, with proportions exceeding 82 wt% and 17 wt%, respectively. Group SM exhibited significantly higher roughness (1.18 μm) than AM0 (0.71 μm) and AM90 (0.51 μm). Group SM exhibited the highest TP values, while groups AM0 and AM90 had statistically similar TP values. AM0 showed the lowest σ value (411.60 ± 73.99 MPa) and larger defects. Groups AM90 and SM (*n* = 15) possessed comparable σ values (969.85 ± 123.13 MPa and 989.72 ± 107.78 MPa, respectively) (p = 0.6417) and Weibull moduli (9.17 and 10.62, respectively).

**Significance:**

SM zirconia showed higher translucency and roughness, while defects reduced translucency in AM zirconia. Flexural strength was lower for AM0 due to larger defects, whereas AM90 matched SM strength.

## 1. Introduction

Additive manufacturing (AM) has emerged as a promising alternative to subtractive manufacturing (SM) for zirconia dental restorations, addressing challenges such as material waste, microfractures, and design limitations inherent in SM [1–9]. Digital light processing (DLP) is an AM technology widely used to create dental ceramic prostheses [10] due to its speed, resolution, precision, and the excellent properties of the resulting ceramic components [2]. This technique employs a slurry composed of ceramic particles and resin monomers which are light-cured according to a computer-aided design (CAD) model [4]. The acquisition of the 3D object occurs through layer stacking and light curing, followed by a debinding and sintering process [2]. The properties of AM zirconia are influenced by various factors, including the build orientation.

The printing orientation during fabrication refers to the specific angle at which the part is placed on the build platform. It is crucial in determining the structural integrity of the final product [11]. Studies have demonstrated that the 0° orientation generally results in higher flexural strength than the 90° orientation [11,12]. However, it is noteworthy that even with a 0° orientation, the flexural strength remains lower than that of subtractively manufactured zirconia [11,13].

Translucency, alongside flexural strength, is a critical property influencing the clinical applications of dental zirconia. Additively manufactured zirconia, with higher porosities and flaw populations, shows lower translucency compared to subtractively manufactured zirconia [13,14]. While AM technology has shown potential for zirconia prostheses, achieving a balance between mechanical strength and optimal translucency remains a challenge. The analysis of key factors such as surface roughness, density, composition, microstructure, and defect populations are essential for understanding and improving the material’s mechanical and optical properties. Roughness and porosity scatter light and weaken the material, while composition such as the tetragonal phase enhances strength through transformation toughening, and the cubic phase improves translucency by reducing light scattering. Defects may act as the origin of fractures and decrease translucency. Further research is needed to establish relationships between optimized printing parameters and these properties with zirconia’s strength and translucency. While flexural strength has been related to defects through fractographic analysis [12,15,16], no prior study has investigated this relationship for the translucency parameter of AM zirconia using SM zirconia as a reference.

This study aimed to investigate the microstructural characteristics of zirconia produced through subtractive and additive manufacturing techniques, with a focus on different build orientations in the additive process, and to establish associations between these characteristics and the material’s flexural strength and translucency properties. The null hypothesis states that there are no significant differences in the microstructure, roughness, density, translucency, or flexural strength of zirconia produced by additive manufacturing in different build orientations compared to zirconia produced by subtractive manufacturing.

## 2. Materials and methods

### 2.1. Specimen fabrication

A disc-shaped specimen (14.00 mm in diameter and 1.20 mm in thickness) was designed using CAD software (Blender, Amsterdam, Netherlands). The resulting standard tessellation language (stl) file was used to fabricate 18 zirconia disc specimens, divided into three groups of six each (*n* = 6). Twelve specimens were fabricated using additive manufacturing. These specimens were fabricated using two different orientations: 6 specimens were printed with a 0° orientation (group AM0), while the other 6 specimens were built with a 90° orientation (group AM90). In the 0° orientation, the layers were built along the central axis of the disc, whereas in the 90° orientation, the layers were stacked in the direction perpendicular to the central axis [11]. Both groups employed lithography-based ceramic manufacturing (LCM) technology, also known as digital light processing or DLP, utilizing the Lithoz Cerafab 7500 Dental 3D printer, using a 3 mol% yttria-stabilized tetragonal zirconia polycrystals slurry (3Y-TZP, LithaCon 3Y 230, Lithoz America, LLC, Troy, NY). Each layer was fabricated with a thickness of 25 μm at a production rate of 70 layers per hour, using a blue light LED with 455 nm wavelength. Each specimen was cleaned with a specialized cleaning solution (LithaSol, Lithoz America, LLC, Troy, NY). The debinding process was carried out at 1000°C, followed by a final sintering process heated up to 1450°C with a dwell time of 2 hours. The manufacturing parameters for all test groups are shown in Table 1.

**Fig. 1.**
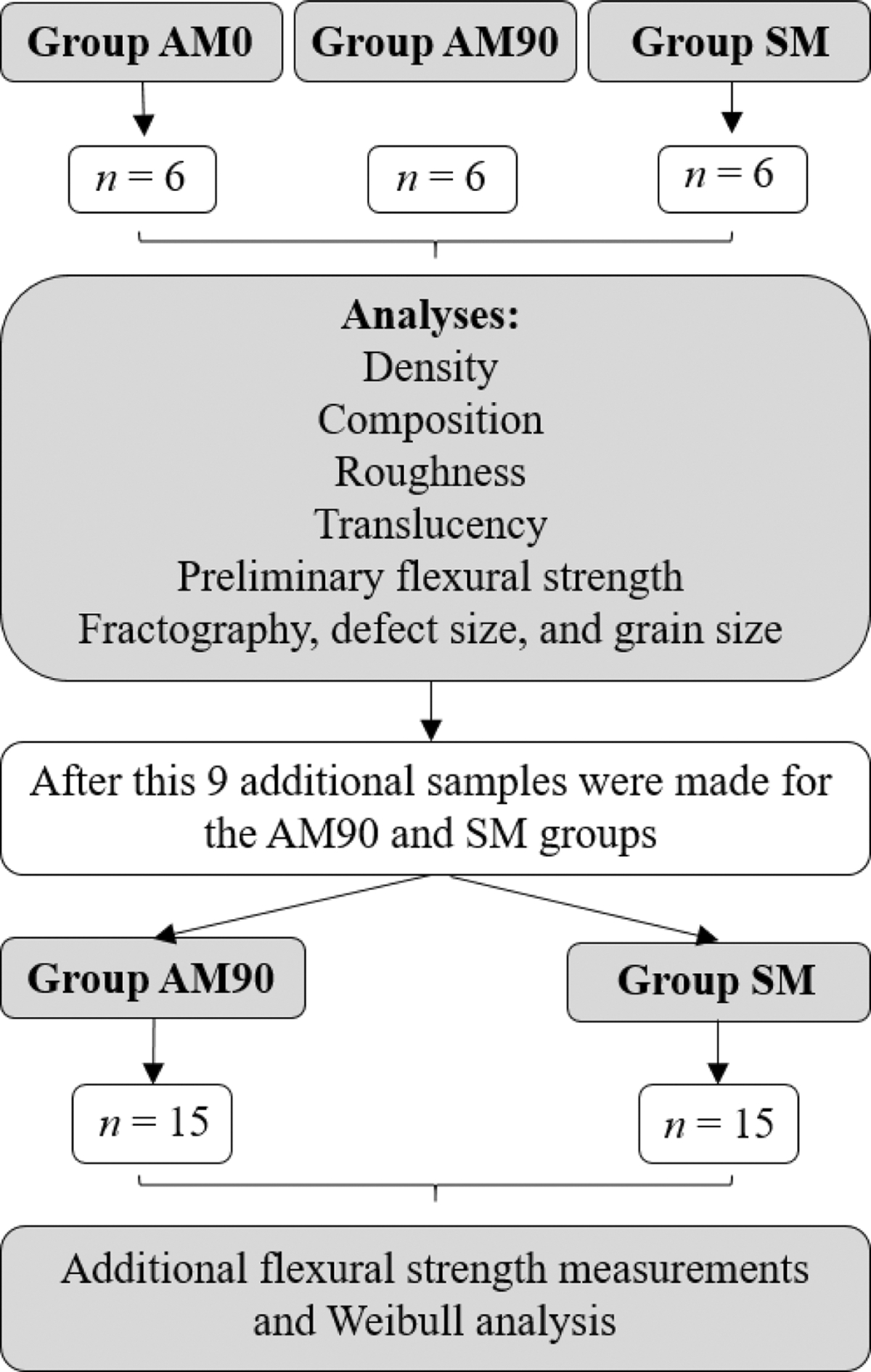
Flowchart of the experimental design.

Six specimens were fabricated using subtractive manufacturing (group SM) from a commercial pre-sintered zirconia CAD/CAM disc (IPS e.max ZirCAD, unshaded, 3 mol% yttria-stabilized tetragonal zirconia polycrystals, 3Y-TZP, Ivoclar Vivadent, Schaan, Liechtenstein), using a five-axis milling machine (model TR5, vhf Inc., Hauppauge, NY). A diamond-coated carbide cutter with a 1 mm diameter and a triple tooth radius design (Z100-R3D-40, vhf Inc.) was employed for the milling of the specimens. Following milling, the specimens were air-dried and cleaned using a soft brush. The sintering process took place in a dental sintering furnace (baSiC Austromat 674, Dekema Dental, Freilassing, Germany), where the specimens were heated up gradually to 1500°C with a dwell time of 2 hours according to the manufacturer’s specifications (Ivoclar Vivadent). These specimens were fabricated to be used in the density, composition, roughness, translucency, preliminary flexural strength, fractography, defect size, and grain size measurements. Fig. 1 shows the flowchart of the study’s experimental design.

### 2.2. Density

The bulk density (BD) of the three groups (*n* = 6) was assessed using the Archimedes water displacement method using an analytical balance equipped with a density determination kit (Mettler Toledo, Columbus, OH, USA) [17]. Relative density (RD) was determined by dividing the experimentally obtained bulk density values by the 3Y-TZP theoretical density value (6.10 g/cm^3^) [18].

### 2.3. Composition

The crystalline phases in sintered zirconia of groups AM0, AM90, and SM specimens were examined by X-ray diffraction (XRD) with CuKα radiation (Rigaku Miniflex 6 G, Tokyo, Japan) (*n* = 3). Specimens were positioned on the specimen stage with the upper side leveled against the flat surface of a rigid slab. Spectra were collected over a 2θ range of 20° – 80° with a step size of 0.01° and a scan rate of 2° per min. The proportions of the tetragonal and cubic phases were determined based on the peak intensities observed in the 2θ range spanning from 72° to 76° [19]. Intensities of the $I_{t}\;(004)$, $I_{t}\;(220)$, and $I_{c}\;(400)$ peaks were utilized for this calculation, following the equation below:

(1) $$X_{c}=\frac{I_{c}(400)}{I_{c}(400)\;+\;I_{t}(004)\;+\;I_{t}(220)}$$

$$X_{t}=\frac{I_{t}(004)\;+\;I_{t}(220)}{I_{c}(400)\;+\;I_{t}(004)\;+\;I_{t}(220)}$$

where $X_{c}$ and $X_{t}$ represent the weight percent of cubic and tetragonal phases, respectively. The $I_{t}$ and $I_{c}$ represent the intensities of the corresponding peaks [20,21].

### 2.4. Roughness

Measurements of surface roughness were taken at the center of each non-polished specimen (*n* = 6) over a 3.3 mm^2^ area using a non-contact optical profilometer (Profilm3D, Filmetrics, CA, USA). Subsequently, the arithmetic mean surface heights (*S*a) and standard deviations were calculated [22].

### 2.5. Translucency

The translucency parameters (TP) for each group (*n* = 6) were measured using a dental spectrophotometer (SpectroShade Micro, MHT, Niederhasli, Switzerland) and color coordinates CIE $L^{*}a^{*}b^{*}$ were measured over standard backgrounds (black $L^{*}=1.8$, $a^{*}=1.3$, $b^{*}=\;-1.5$ and white $L^{*}=95.7$, $a^{*}=-1.3$, $b^{*}=2.6$) [23]. A drop of coupling liquid (refractive index: 1.8, Gem Refractometer Liquid, Cargille Laboratories, Inc., Cedar Grove, NJ, USA) was applied between the specimen and background to ensure optical continuity [24]. The TP values were determined by the color difference between the specimen on black (B) and white (W) backgrounds, according to equation:

(2) $$TP=\sqrt{{\left(L_{B}^{*}-L_{W}^{*}\right)}^{2}+{\left(a_{B}^{*}-a_{W}^{*}\right)}^{2}+{\left(b_{B}^{*}-b_{W}^{*}\right)}^{2}}$$

where $L^{*}$, $a^{*}$, and $b^{*}$ refer respectively to the lightness, redness to greenness, and yellowness to blueness coordinates in the CIE color space [25].

### 2.6. Preliminary flexural strength

Biaxial flexural strength was determined using the piston-on-3-ball method. For preliminary study, 6 specimens of each group were tested. The loading piston and support balls were constructed from hardened steel, with diameters of 1.40 mm and 3.20 mm, respectively, in compliance with the International Organization for Standardization (ISO/FDIS 6872:2024) guidelines [26]. To minimize friction, Teflon tape was positioned between the support balls and the specimens, while Scotch tape was applied between the loading piston and the specimens to prevent contact-induced damage. The specimens (Ø14.00 × 1.20 mm) were loaded monotonically at a rate of 1.00 mm/min in a universal testing machine (model 68TM-5, Instron, Norwood, MA) until catastrophic fracture occurred. The biaxial flexural strength of each specimen was then calculated in Megapascals (MPa) using Eq. 3, following ISO/FDIS 6872:2024 [26].

(3) $$\sigma =\frac{-0.2387p(X\;-\;Y)}{d^{2}}$$

where

$$X=(1+v)ln{\left(r_{2}/r_{3}\right)}^{2}+[(1-v)/2]{\left(r_{2}/r_{3}\right)}^{2};$$

$$Y=(1+v)[1+ln{\left(r_{1}/r_{3}\right)}^{2}]+(1-v){\left(r_{1}/r_{3}\right)}^{2}$$

where $r_{1}=5.00\;mm$ (radius of support circle), $r_{2}=0.70\;mm$ (radius of the loaded area), $r_{3}=7.00\;mm$ (disc radius), b=1.20mm (disc thickness), v=3.15 (Poisson’s ratio for 3Y-TZP) [27], and p is the maximum load the specimen can withstand before a fracture occurs (N).

The results of this preliminary flexural strength test were utilized for exploratory purposes to identify the optimal printing orientation (0° or 90°). Subsequently, a set of 15 specimens was prepared to conduct additional biaxial flexural strength testing for Weibull analysis.

### 2.7. Fractography, defect size, and grain size

An extensive post-mortem fractographic analysis was conducted to identify the fracture origins and analyze the microstructural characteristics of zirconia specimens manufactured by AM and SM techniques. Specimens were first analyzed using a polarized light microscope (AxioZoom V.16, Zeiss, Oberkochen, Germany) and further subjected to qualitative fractographic analysis using scanning electron microscopy (SEM) (Quanta 600 FEG ESEM) [28]. In addition, critical defect size was estimated based on Eq. 4 [28]:

(4) $$c_{\text{calc}\;}={\left(\frac{K_{IC}}{Y\sigma_{F}}\right)}^{2}$$

where $K_{IC}$ is the fracture toughness, $\sigma_{F}$ is the fracture stress at the origin location, Y is the geometric factor of stress intensity related to the defect geometry, and $c_{\text{calc}}$ is the calculated defect size. The $K_{\text{IC}}$ used was 4.7 MPa√m and Y was 1.13 [28].

The grain size measurement involved examining a minimum of 500 grains in one representative specimen, using ImageJ software and the arithmetic mean was calculated (U.S. National Institutes of Health, Bethesda, Maryland, USA) [29].

### 2.8. Additional flexural strength measurements and Weibull analysis

After evaluating the results from preliminary flexural strength tests, fractography, and defect size analysis, it was observed which printing orientation (0° or 90°) yielded superior outcomes in terms of both mechanical performance and microstructure. Consequently, an additional 9 specimens were manufactured for the optimal printing orientation in additive manufacturing (group AM90), and 9 more specimens were fabricated for the subtractive group (SM), in addition to those previously fabricated, culminating in two groups comprising 15 specimens each (*n* = 15), in accordance with ISO/FDIS 6872:2024 [26]. This ensured the minimum required specimen size for flexure testing and allowing for subsequent Weibull analysis [26]. The flexural strength of the AM90 and SM groups was analyzed using the same methodology described in the “Preliminary flexural strength” section.

To predict the fracture resistance of the AM90 and SM groups, the Weibull failure probabilities were calculated. The Weibull modulus, m, characterizes the failure probability according to the Weibull distribution. A greater m value indicates a reduced dispersion in the measured strength of zirconia. For critical flexural stress (strength) $\sigma_{F}$ of zirconia discs, the Weibull failure probability P can be defined as:

(5) $$P=1-exp\left[-{\left(\sigma_{F}/\sigma_{0}\right)}^{m}\right]$$

where $\sigma_{0}$ is a scaling stress. For a data set of critical stresses, cumulative probabilities are calculated by ranking values in ascending order and evaluating corresponding $\sigma_{F}$ values. A plot of ln(ln(1/(1-P))) against $ln\;\sigma_{F}$ gives a straight line with slope m:

(6) $$ln(ln(1/(1-P)))=m\left(ln\;\sigma_{F}-ln\;\sigma_{0}\right)$$

The Weibull curves presented in this study were derived by analyzing the scatter plot of cumulative probability versus logarithm of measured strength data. This analysis involved employing the least-squares method for linear regression, with a 95 % confidence interval, to fit the data. The slope of the line of best fit represents the Weibull modulus, m.

### 2.9. Statistical analysis

An one-way ANOVA test was used to compare mean values between groups followed by Tukey’s post hoc test for translucency, density, roughness, and preliminary flexural strength. An independent samples *t*-test was used to compare mean values between groups for the flexural strength test. The obtained Weibull moduli m were statistically compared between the groups and the differences between groups were identified based on the non-overlap of the 95 % two-sided confidence interval (CI). The statistical significance level was set at to α = 0.05.

## 3. Results

### 3.1. Density

Groups AM0 and AM90 showed a bulk density of 6.07 ± 0.01 g/cm^3^ and 6.07 ± 0.02 g/cm^3^, respectively, and similar to the SM group which was 6.08 ± 0.01 g/cm^3^ (Table 2). For the three groups, the relative density was higher than 99.50 %. No statistically significant differences were found among the groups (p < 0.05).

### 3.2. Translucency

The SM group exhibited the highest value for the translucency parameter (%), 17.00 ± 0.84, which was statistically different from both AM groups (p < 0.05). AM0 presented a TP value of 8.64 ± 0.50, while AM90 had a value of 9.34 ± 0.41 (Table 2).

### 3.3. Roughness

**Fig. 2.**
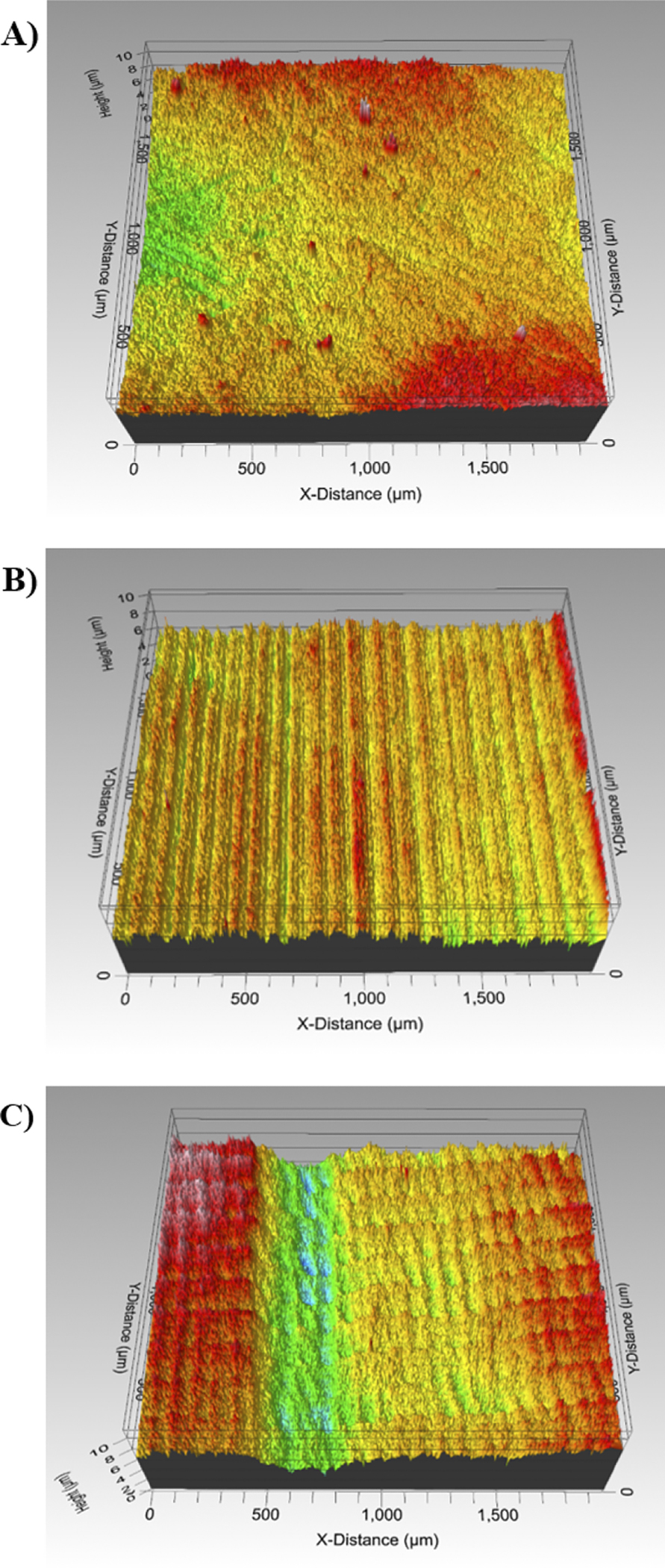
Pattern of surface roughness (A) AM0 group, (B) AM90 group, and (C) SM group.

The SM group had the highest roughness *S*a value, 1.18 ± 0.13 μm, while AM0 and AM90 obtained values of 0.71 ± 0.09 μm and 0.51 ± 0.10 μm, respectively (Fig. 2). There was no statistical difference between the AM groups, but both were different from SM (p < 0.05) (Table 2).

### 3.4. Composition and grain size

All groups comprised around 83 % by weight of the tetragonal zirconia phase (*t*-ZrO_2_) and approximately 17 % by weight of the cubic phase (*c*-ZrO_2_). This similarity arises from the fact that both the LithaCon 3Y 230 (AM0 and AM90) and IPS e.max ZirCAD (SM) materials were made using 3Y-TZP grade zirconia raw powders sourced from Tosoh Corp., Tokyo, Japan. The grain size estimation was calculated in a specimen of the AM90 group and resulted in an average grain size of 0.48 μm, similar to the values reported in the literature for subtractively manufactured zirconia [30,31].

### 3.5. Preliminary flexural strength data

Group AM0 showed the lowest flexural strength σ of 411.60 ± 73.99 MPa, whereas AM90 and SM groups had a flexural strength of 970.10 ± 42.01 MPa and 1021.00 ± 77.14 MPa, respectively (Table 2). No statistically significant difference was found between the AM90 and SM groups, however, group AM0 was statistically different from both groups (p < 0.05).

### 3.6. Fractography and defect size

**Fig. 3.**
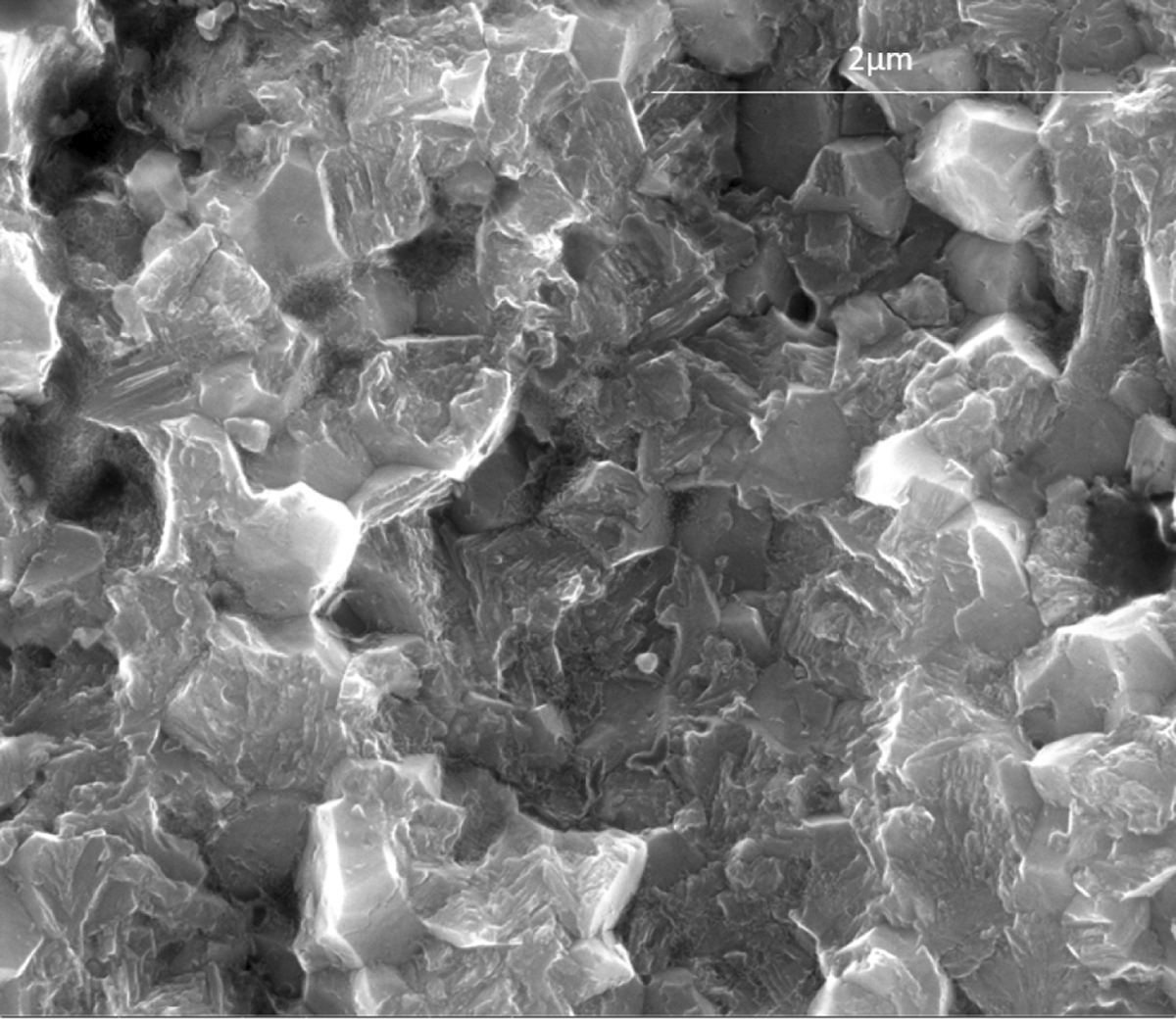
Predominant intragranular fracture pattern found in all groups.

**Fig. 4.**
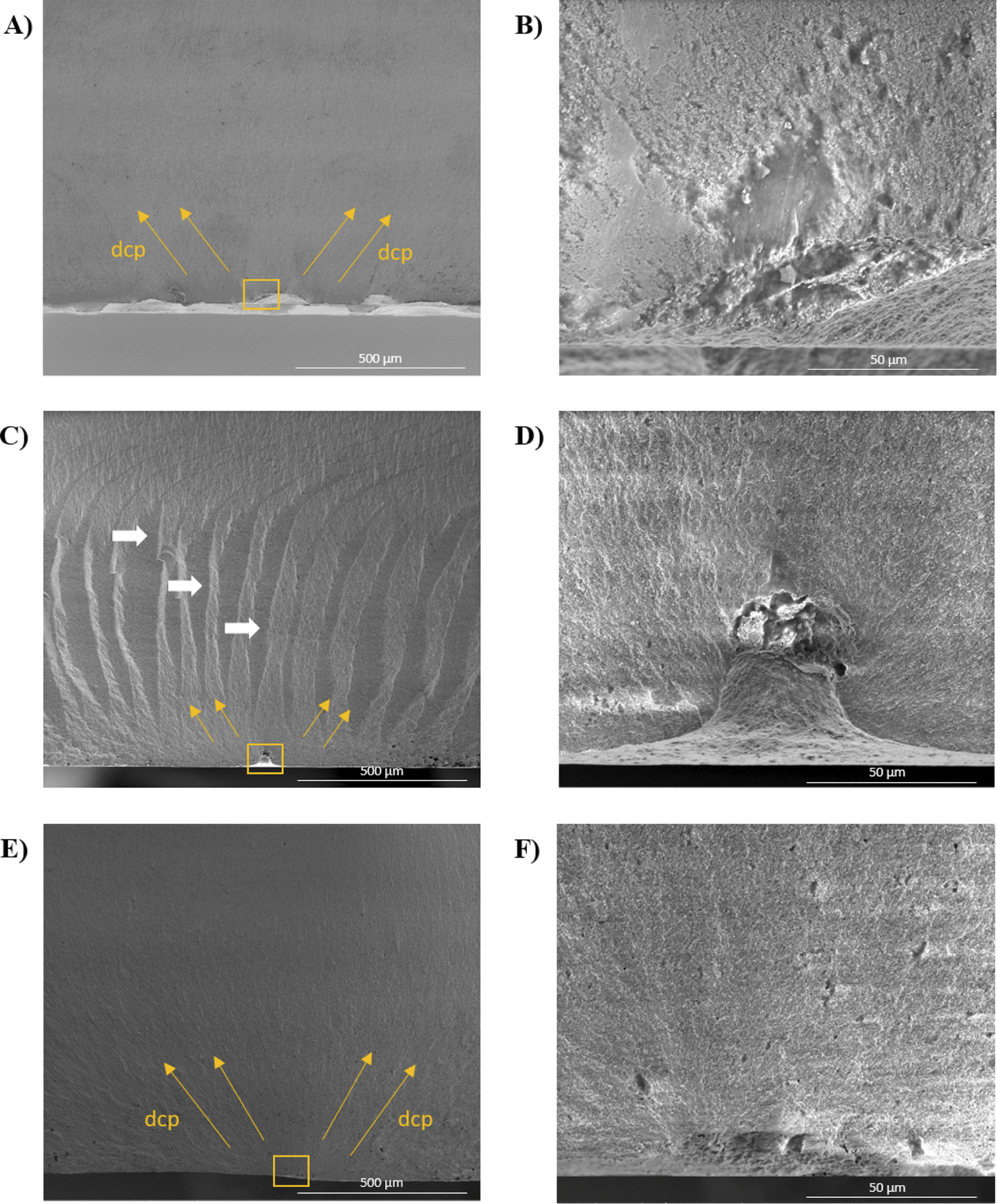
Presents specimens from various groups, each revealing distinctive characteristics upon closer examination. (A) In the AM0 group, a fracture originates from a void surrounded by porosities. (B) A closer inspection of the same AM0 specimen, the fracture origin becomes evident—a void surrounded by porosities. (C) In the AM90 group, delamination between layers is apparent, as indicated by white arrows. (D) Upon closer examination of the AM90 specimen, an agglomerate emerges as the origin of the fracture. (E) In the SM group, a fracture is observed, with the defect introduced by the milling tool evident upon closer inspection of the same specimen in (F). Notably, squares pinpoint the origin of the fracture in each specimen, while yellow arrows indicate the direction of crack propagation (dcp).

**Fig. 5.**
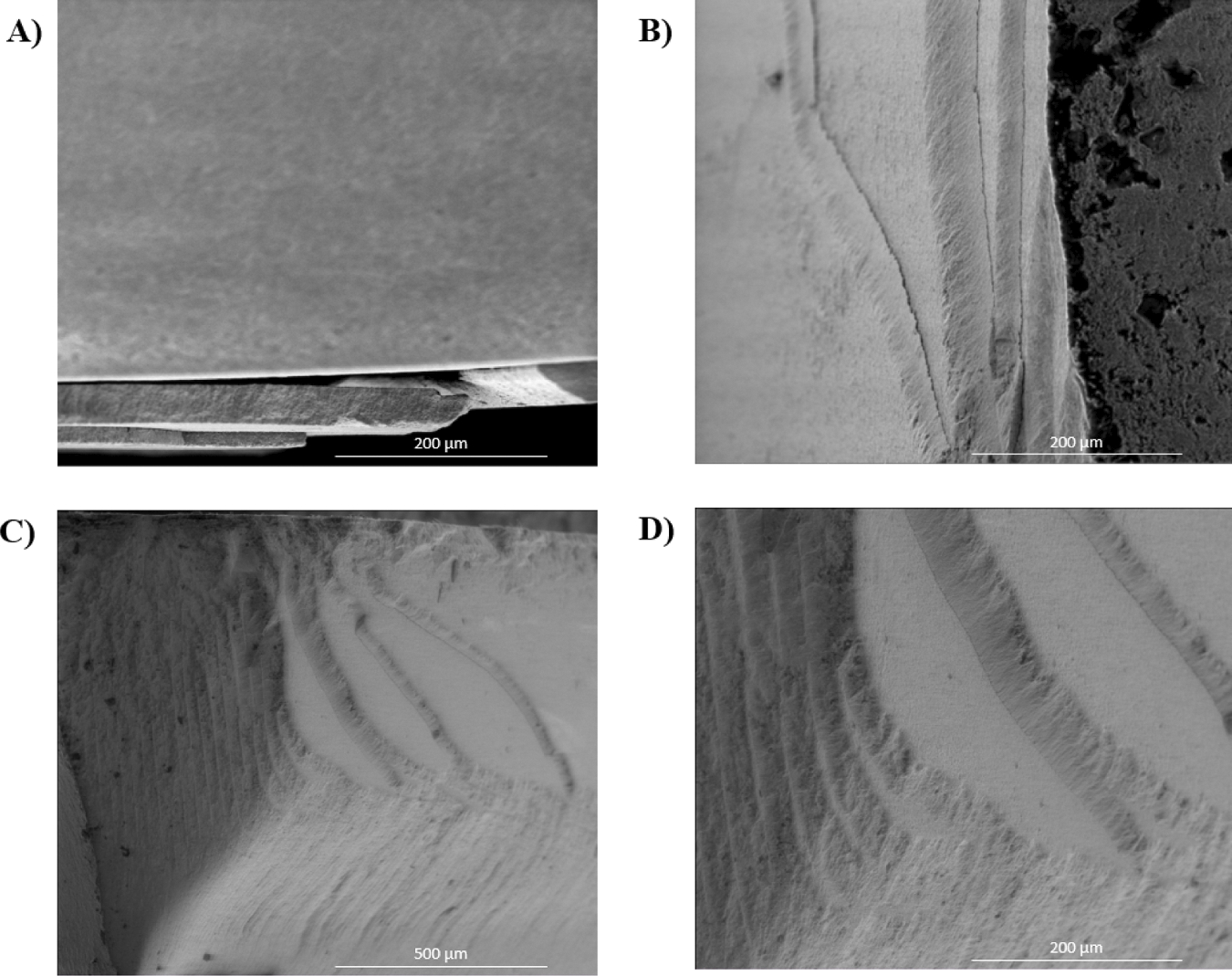
Delamination occurring within the layers of both the AM0 and AM90 groups. (A) Layers delamination in the AM0 group, while (B) showcases a similar phenomenon in the AM90 group; (C) and (D) images are layers delamination in the AM90 group, with (D) offering a higher magnification of the same spot.

**Fig. 6.**
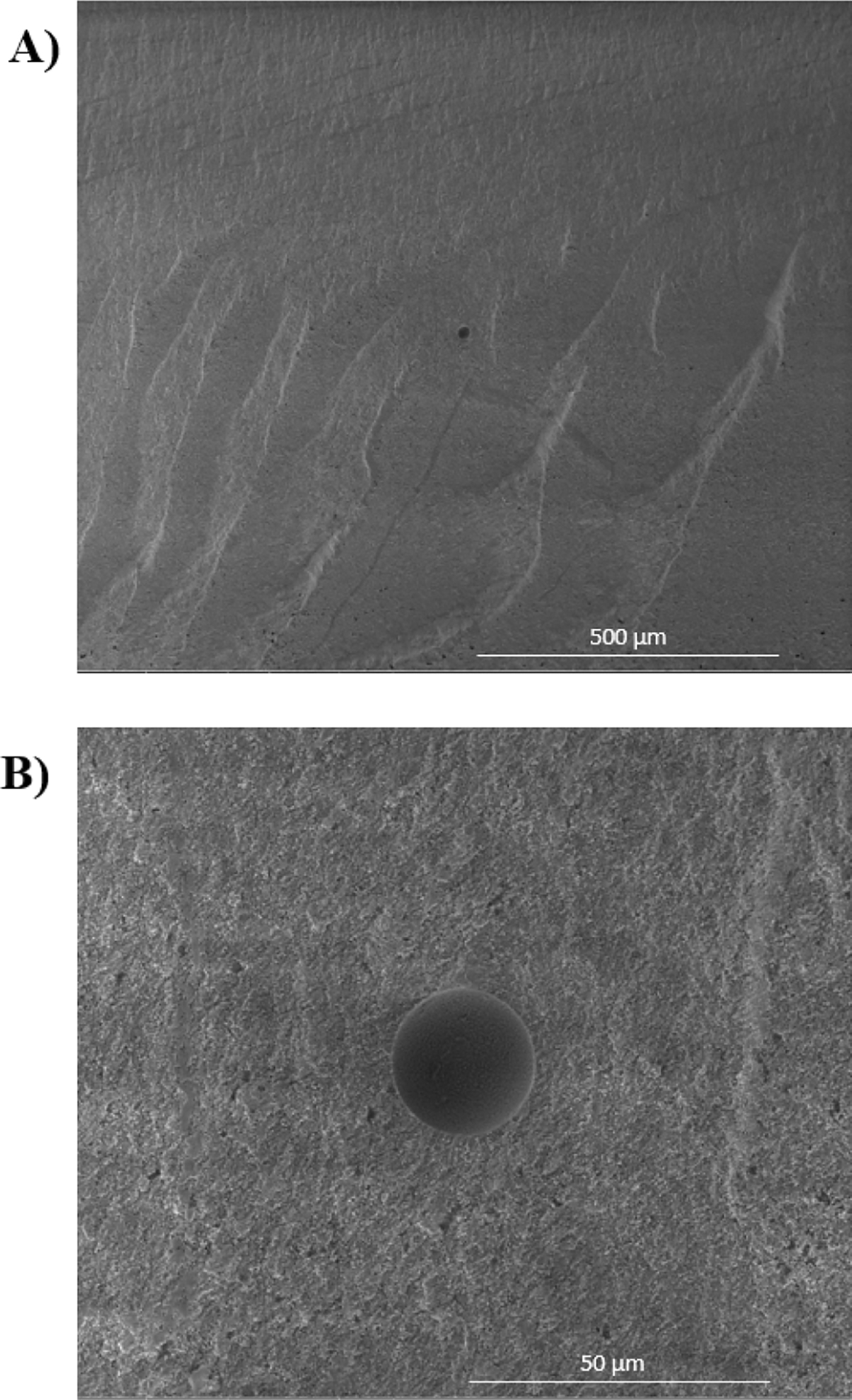
Both (A) and (B) provide lower and higher magnifications of the same spot, offering a comprehensive view of a pore trapped within the microstructure of an AM90 specimen.

The morphological examination of fractured surfaces across all experimental groups consistently revealed a predominant intragranular fracture mode, evident at the fracture origin and its vicinity that extended to almost 600 μm from the origin (Fig. 3). Fractography analysis showed that fracture always started at the tensile surface of the specimens and the critical defects were located near the tensile surface in all groups (Fig. 4). Voids/pores and agglomerates were identified as the origins of the fractures for the AM groups and the SM group. In addition, milling-induced defects were also identified in the latter group. The critical defect size was calculated (Eq. 4) and the group AM0 presented the largest values, ranging from 20.24 μm to 52.26 μm, relative to the AM90 and SM groups, which exhibited critical defect sizes ranging from 4.33 μm to 10.81 μm and 3.94 μm to 11.14 μm, respectively. While the critical defect size calculation is inherently an estimation, validation of the accurate identification of the failure origin can be achieved through comparative analysis between the estimated value and the defect size ascertained via fractographic analysis utilizing SEM. In the present study, the critical defect sizes identified in the SEM images corroborated with the estimated critical defect values. Furthermore, extensive porous regions were found in the specimens obtained through additive manufacturing (Fig. 4B); additionally, delamination between printing layers was identified in both the AM0 and AM90 groups (Fig. 5). Also, porosities were observed in the microstructure of the AM groups (Fig. 6). Interestingly, despite the substantial size of these pores, with some even surpassing the estimated critical defect size, they were located far from the tensile surface and did not act as the fracture origin.

### 3.7. Flexural strength and Weibull modulus

**Fig. 7.**
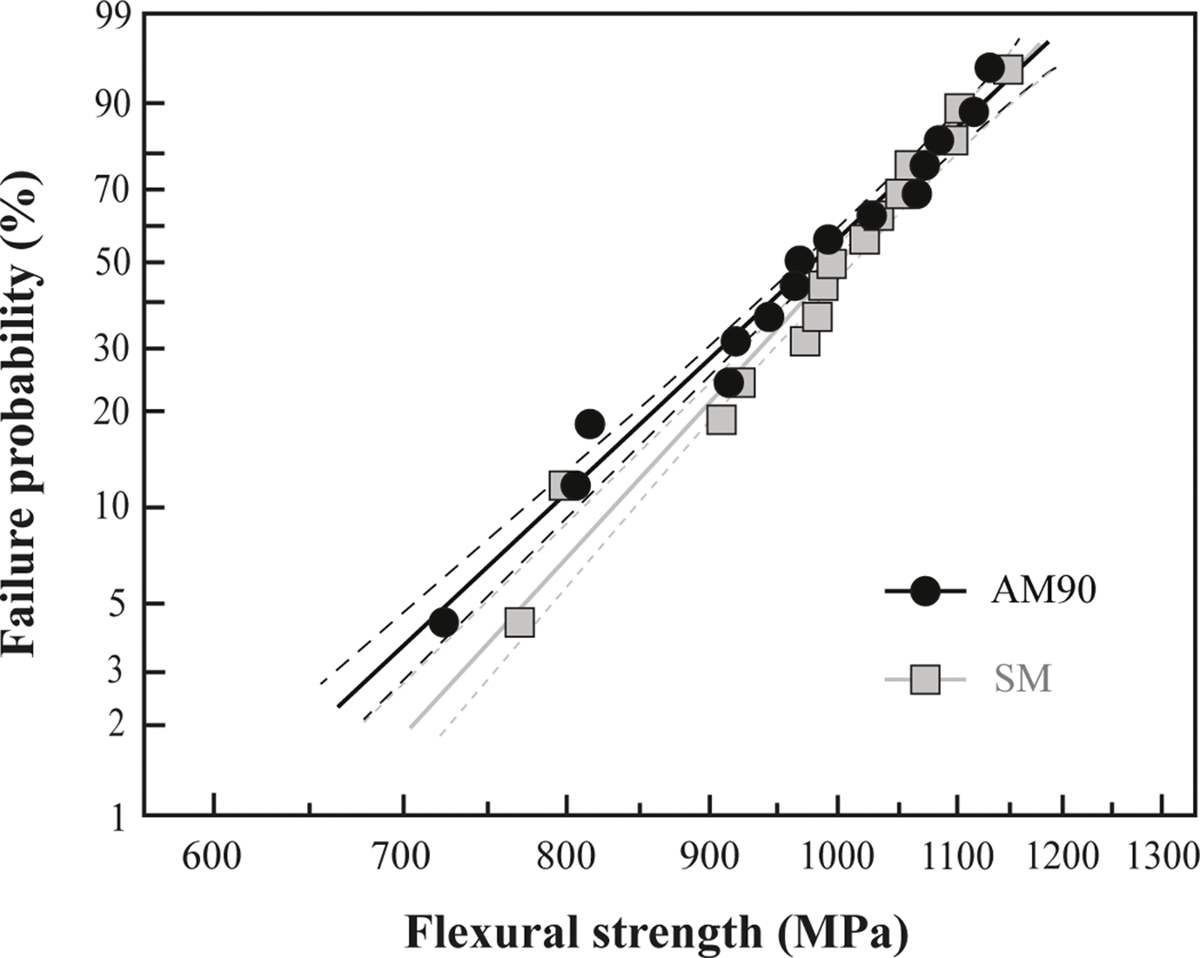
Flexural strength of AM90 (black circles) and SM (grey squares) groups. Black dashed curves and grey dashed curves represent 95 % confidence intervals for the AM90 and SM data sets, respectively.

The findings from the preliminary assessments of flexural strength, fractography, and defect size consistently indicated that the 90° printing orientation resulted in superior mechanical properties and microstructure relative to the 0° printing orientation. Consequently, additional flexural strength tests were conducted to compare additively manufactured specimens (group AM90) printed at a 90° orientation with subtractively manufactured zirconia (SM). Group AM90 demonstrated a flexural strength value of 969.85 ± 123.13 MPa, similar to that of 989.72 ± 107.78 MPa exhibited by group SM, with no statistically significant difference observed between the two groups (p = 0.6417) (Table 3 and Fig. 7). The Weibull modulus m for the AM90 group was 9.17, whereas for the SM group, it was 10.62, with no statistically significant difference between the two groups.

## 4. Discussion

Despite extensive research efforts aimed at identifying optimal printing parameters for zirconia, the literature currently lacks a consensus on the most effective protocols to consistently achieve superior performance [10]. Thus, this study evaluated the microstructure of additively manufactured zirconia in different build orientations and established relationships between flexural strength and translucency. A subtractively manufactured zirconia was used as a reference. The null hypothesis states that there would be no significant differences in the microstructure, roughness, density, translucency, or flexural strength of zirconia produced by additive manufacturing in different build orientations compared to zirconia produced by subtractive manufacturing was partially rejected.

Despite a slight difference in the sintering protocol between the zirconia groups obtained by AM and SM, this difference was not sufficient to affect the density, as no statistically significant difference was observed between the groups. It is important to highlight that the sintering protocol was conducted according to the manufacturers’ recommendations. Therefore, the protocol suggested by each manufacturer appears to be appropriate to ensure that both manufacturing methods yield high and adequate densities (>99.5 % for all groups). Our study aligns with the findings of Tan X, et al. (2022) [32], who evaluated both conventional and high speed sintering protocols for zirconias produced using AM and SM technologies. Their study, conducted at temperatures very similar to those used in our research, also reported no statistically significant differences between the groups, with high densities (>99.2 %) for all groups.

Regarding the roughness analysis, the SM group displayed the highest *S*a values among all groups. The higher roughness of the SM group can be attributed to the U-shaped grooves left on the specimen surface by the milling burs [22], as observed in the roughness images, indicating that the groove dimensions were likely influenced by the dimensions of the fluted tungsten carbide milling tool. Our results corroborate with the findings of Zandinejad et al. [33], who reported that the surface roughness of AM (0.61 μm) zirconia was statistically lower than that of SM zirconia (1.65 μm). However, our findings partially align with those of Abualsaud et al. [11], who also observed similar values for AM zirconia in both printing directions (0.63 μm for 0° and 0.66 μm for 90°), while the SM group exhibited values statistically comparable to those of the AM groups (0.54 μm). Usually, lower roughness correlate with improved translucency. However, the SM group exhibited the highest translucency values among all groups. This can be explained because, the lower translucency observed in the AM groups, which is not suitable for clinical use in the aesthetic zone, is attributed to porosities and defects within their microstructure [13,31]. As evidenced by the fractography analysis, the choice of printing parameter, particularly the printing orientation, can significantly influence the size of defects in the specimens, which in turn can impact the flexural strength values, especially when the defects are located near the tensile surface of the specimen. Nonetheless, it is important to emphasize that, regardless of the size of defects or pores, they invariably affect the translucency of 3Y-TZP, even in small submicron dimensions [31]. This phenomenon arises from the disparity in refractive indices between zirconia and air, leading to light scattering at pores and thereby reducing translucency [31].

Another noteworthy aspect that could have influenced the translucency parameters is the inadequate adherence of layers in specimens fabricated by additive manufacturing, which has been confirmed by fractographic analysis revealing delamination between the printing layers. This issue may further exacerbate light scattering at the interface of the layers and decrease translucency when compared to zirconia produced via subtractive methods, despite the high density found in all groups. Factors associated with the additive manufacturing method, such as light source and wavelength utilized, filler content, ink viscosity, and debinding and sintering protocols, may all contribute to zirconia translucency [34–39]. A few studies have investigated these parameters in an effort to achieve both good mechanical properties and high translucency simultaneously in printed zirconia [34–39]. A notable demonstration of this is the fact that the zirconia slurry used for additive manufacturing typically has a high viscosity, and the DLP technology involves layering the specimen. This may result in porosities appearing during the printing process. These pores are present within the structure and through the layers and may not be easily eliminated during the sintering process, as evidenced by the fractographic findings of this study.

Our findings corroborate partially with a singular study that has investigated the translucency of printed zirconia with different build directions, exhibiting better results for 90° orientation compared to 0° orientation [14]but both lower than subtractive manufacturing. Also, the present findings corroborate with another study [13] from our research group that found statistical differences between additively and subtractively manufactured zirconia in terms of translucency, favoring the subtractive group. Considering this, it is evident that there remains significant untapped potential for further exploration of this technology, especially in terms of optical properties.

In the preliminary flexural strength test (Section 3.5), there was a significant difference between group AM0 and the other AM90 and SM groups. The flexural strength of the AM0 group failed to meet the requirements outlined by ISO/FDIS 6872:2024 for fabricating either a monolithic or layered substructure for a three-unit prosthesis, particularly one involving a molar, which requires a flexural strength of 500 MPa [26]. Conversely, the AM90 group not only fulfilled ISO criteria but also demonstrated comparable flexural strength to the SM group, exhibiting no statistically significant differences. This outcome is attributed to the identification of critical defects, with the AM0 group having larger defects compared to the other groups. The 0° printing orientation resulted in larger defects than the 90° orientation due to its larger print areas and shorter distance between the bottom and top planes. The inherently high viscosity of the zirconia slurry has a greater tendency to form air bubbles and defects over large areas. With relatively weak interfaces between the layers, these pores and defects could propagate through multiple layers, forming strength-limiting flaws. This observation corroborates with findings from Harrer et al. (2017) [15], who similarly noted diminished strength values and increased defect sizes in specimens printed at 0° compared to those printed at 90°. It is noteworthy that they utilized the same printing machine employed in the current study.

In the flexural strength test (Section 3.7), both the AM90 and SM groups displayed highly comparable results, with no differences between them, as expected based on the preliminary flexural strength test. Furthermore, the density of these two groups was nearly identical, and their phase compositions exhibited remarkable similarity. This outcome was anticipated since the raw zirconia powder used in both groups originated from the same source (Tosoh Corp., Tokyo, Japan). Although AM zirconia usually displays elevated porosity, previous investigations have suggested that a moderate degree of porosity may not markedly compromise the strength of 3Y-TZP, provided that the size of the pores remains within the bounds of the strength-limiting flaws [40]. The Weibull modulus, denoted as m, measures the variability within the results, with higher values indicating more uniform distributions of defect sizes [41]. In this study, no statistical difference was observed between the AM90 and SM groups in terms of the Weibull modulus. Interestingly, Zenthofer et al. ¨ [42] and Rues et al. [43] found no significant difference in flexural strength between the 90° orientation and subtractively manufactured zirconia, which is consistent with our findings. Notably, the zirconia brands and the printer used in their studies were the same as those used in this study. However, other studies have shown that the 0° orientation generally results in higher flexural strength than the 90° orientation [11,12]. In the study by Abualsaud et al. [11], although the 0° orientation demonstrated better results than the 90° orientation, it is important to note that the zirconia brand and printer used were different from those in our study. While Marsico et al. [12] used 5Y-PSZ, in contrast to the 3Y-TZP used in this research, higher strength values were observed in the 0° orientation relative to the 90° orientation. On the other hand, some studies have reported that the SM group exhibited higher flexural strength than the AM samples printed in the 0° orientation [44,45], which is consistent with our findings. This highlights the need for further studies to define the optimal protocol for dental zirconia printing.

The results of the present study demonstrate promising outcomes for the use of AM zirconia. Nevertheless, further developments are still required. This study has several limitations such as utilizing one layer height and one type of sintering and debinding protocol, as well as utilizing only one printing resolution. Therefore, additional research is warranted in this field, exploring various parameters to identify the most suitable ones for the application of AM dental zirconia.

## 5. Conclusions

Given the limitations of the present study, the following conclusions can be drawn from the analyses conducted on density, composition, roughness, translucency, flexural strength, fractography, and defect size:

- There was no difference in the density of zirconia specimens additively manufactured in different print directions (0° and 90°) relative to their subtractively manufactured counterparts.
- Subtractively manufactured zirconia exhibits higher translucency parameters and roughness compared to additively manufactured zirconia.
- Large voids and defects have a detrimental effect on the translucency of additively manufactured zirconia.
- In terms of flexural strength, additively manufactured zirconia in the 0° print orientation exhibited lower values and larger defects, whereas those printed in the 90° orientation showed comparable strength and defect sizes to subtractively manufactured zirconia.
- The size and location of defects influence the flexural strength of dental zirconia.

## Figures and Tables

**Table 1:** Manufacturing parameters for AM and SM technologies.

Group	AM0	AM90	SM

Classification by Y_2_O_3_ (%)	3Y-TZP		3Y-TZP
Commercial material brand	LithaCon 3Y 230, Lithoz		IPS e.max ZirCAD, Ivoclar
Manufacturing machine	Lithoz CeraFab 7500 ceramic 3D printer		Five-axis milling machine, TR5, vhf Inc.
Printing orientation	0°	90°	-
Layer thickness	25 μm		-
Light source	Blue light LED with 455 nm wavelength		
Exposure intensity	100 mW/cm^2^		-
Exposure energy	190 mJ/cm^2^		-
Estimated time per layer	51 seconds		-
Fabrication speed	70 layers/hour		8 – 10 minutes per specimen

**Table 2:** Overview of the results for tests comparing AM0, AM90, and SM groups. Values are presented in mean and standard deviation.

	AM0	AM90	SM

Bulk Density, ρ (g/cm^3^)	6.07 ± 0.01 ^A^	6.07 ± 0.02 ^A^	6.08 ± 0.01 ^A^
Relative Density, RD (%)	99.55 ± 0.21 ^A^	99.51 ± 0.31 ^A^	99.70 ± 0.16 ^A^
*t*-ZrO_2_ (wt%)	82.56 ± 0.28 ^A^	82.98 ± 0.01 ^A^	82.69 ± 0.10 ^A^
*c*-ZrO_2_ (wt%)	17.44 ± 0.28 ^A^	17.02 ± 0.01 ^A^	17.31 ± 0.10 ^A^
Roughness, *S*a (μm)	0.71 ± 0.09 ^A^	0.51 ± 0.10 ^A^	1.18 ± 0.13 ^B^
Translucency Parameter, TP (%)	8.64 ± 0.50 ^A^	9.34 ± 0.41 ^A^	17.00 ± 0.84 ^B^
Preliminary Flexural Strength, σ (MPa)	411.60 ± 73.99 ^A^	970.10 ± 42.01 ^B^	1021.00 ± 77.14 ^B^

Mean values followed by different uppercase letters indicate significant differences between groups (p < 0.05).

**Table 3:** Biaxial flexural strength and Weibull modulus results comparing AM90 and SM groups. Values are presented in mean and standard deviation, except for Weibull modulus, which is presented in mean and two-sided confidence interval.

	AM90	SM

Biaxial flexural strength, *σ* (MPa)	969.85 ± 123.13 ^A^	989.72 ± 107.78 ^A^
Weibull modulus, *m*	9.17 ^A^	10.62 ^A^

Mean values followed by different uppercase letters indicate significant differences between groups (p < 0.05).

Figure captions
